# Using Noninvasive Genetic Sampling to Survey Rare Butterfly Populations

**DOI:** 10.3390/insects10100311

**Published:** 2019-09-23

**Authors:** Caroline Storer, Jaret Daniels, Lei Xiao, Kristin Rossetti

**Affiliations:** 1 McGuire Center for Lepidoptera and Biodiversity, Florida Museum of Natural History, 3215 Hull Road, Gainesville, FL 32611, USA; cgstorer@ufl.edu (C.S.); lxiao@floridamuseum.ufl.edu (L.X.); khyla@ufl.edu (K.R.); 2 Department of Entomology and Nematology, University of Florida, 1881 Natural Area Drive, Steinmetz Hall, University of Florida, Gainesville, FL 32611, USA

**Keywords:** noninvasive, nonlethal, Lepidoptera, conservation, population genetics, endangered, chorion, *Cylcargus thomasi bethunebakeri*, *Callophrys irus*

## Abstract

Advances in nondestructive genetic sampling techniques continue to offer new opportunities for studying organisms, particularly those of conservation concern where more traditional invasive sampling methods are often not available. As part of a proof-of-concept, we investigated the effectiveness of using the chorion from residual butterfly egg debris as a source of viable genetic material for analysis. Laboratory material from a captive breeding population of the federally endangered Miami blue butterfly (*Cyclargus thomasi bethunebakeri*) was used to test efficacy and refine the methodology. The resulting best practices were subsequently evaluated using field-collected material from extant north Florida populations of the at-risk frosted elfin butterfly (*Callophyrs irus*). Our results demonstrated that it is possible to extract DNA of sufficiently high quantity and quality for successful gene sequencing. We additionally describe a simple, low-cost, and reliable method of collecting and storing egg debris samples that can be consistently adopted for field or laboratory work as well as deployed with projects that have a larger geographic scope and/or involve citizen scientists. Potential limitations related to field sample collection are discussed as well as needs for future evaluation.

## 1. Introduction

Recent advances in molecular techniques continue to offer new, unprecedented opportunities to increase our understanding of organisms without subjecting them to lethal harm [1,2,3,4,5]. The use of noninvasive genetic sampling offers tremendous utility for conservation practitioners to help inform management and recovery decision-making by securing high yield and quality DNA from rare or imperiled taxa. For many vertebrate classes, noninvasive genetic sampling has generally included collecting feces, urea, hair, shed skin, or molted feathers among other sources [6,7,8,9,10]. For many arthropods, specifically insects however, the predominant available options have often involved the removal of wing clips, palps, leg clips, or individual legs [11,12,13,14,15,16,17]. Despite being nonlethal, such methods still require capture and handling of the target organism which can inflict significant stress, inadvertent damage, or death, and may not be feasible for low density or at-risk populations. Alternatively, researchers have also successfully secured DNA from frass, exuviae, and hemolymph in defensive secretions [18,19,20,21,22]. However, these sources may not be broadly available for many taxa and certainly offer significant challenges for reliable collection in the field. Here, we investigated the effectiveness of using the chorion from hatched ovae of the federally endangered Miami blue butterfly (*Cyclargus thomasi bethunebakeri*) (Lepidoptera: Lycaenidae) for DNA extraction and analysis. We additionally provide a validated protocol for field collection with the closely related *Callophrys irus irus*. This taxon is univoltine throughout its range. In Florida, *C. irus irus* is associated with fire-maintained sandhill pine and oak upland habitat that support its sole larval host, *Lupinus perennis*.

## 2. Materials and Methods

### 2.1. Samples

**Figure 1:**
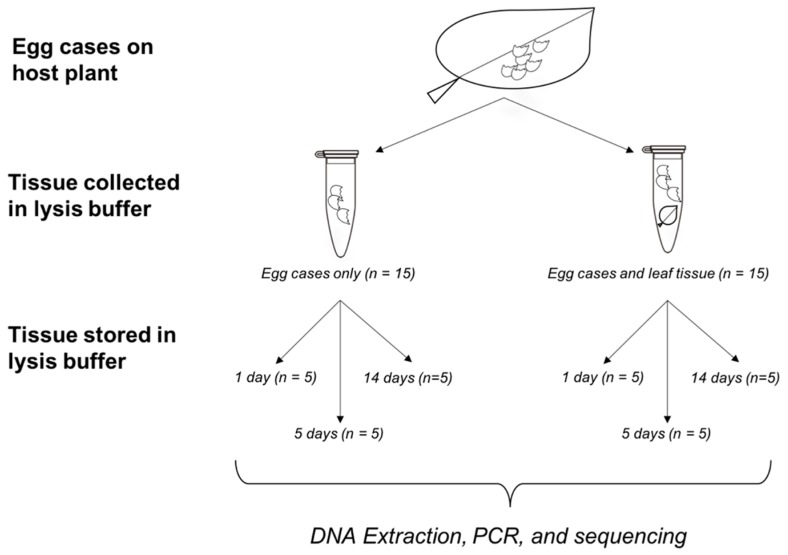
Schematic of proof-of-concept study design.

As part of our proof-of-concept for noninvasive genetic sampling of rare populations, we took advantage of material readily available from a captive breeding population of *C. thomasi bethunebakeri* maintained at the McGuire Center for Lepidoptera and Biodiversity, Florida Museum of Natural History in Gainesville, Florida, U.S.A. The primary objective was to determine if sufficient genetic material for gene sequencing could be recovered from egg case debris left by recently hatched larvae. Secondarily, we were also interested in the effects of non-target material carryover, such as host plant tissue, when target DNA is rare or in low-abundance. Non-target tissue carryover can reduce yields of rare target DNA, especially if non-target tissue is fresher and relatively more abundant. Because these butterfly egg cases are small (~1 mm), delicate, and potentially low quality, non-target tissue carryover is likely and possibly unavoidable. To address this concern, sampling conditions included variable source material: egg cases only (n = 15) or egg cases + host plant tissue (n = 15; Figure 1). Treatments with host plant tissue received a hole punch size circle approximately 1 cm in diameter. Five of each of these source material combinations was randomly assigned to a storage treatment in lysis buffer at room temperature for 1, 5, or 14 days prior to DNA extraction (Figure 1). When collecting egg case samples, time since hatching was not controlled for and ranged between 1 and 10 days. This uncertainty in time since hatching reflects that of field conditions where this variable is often unknown. Initial attempts to prepare egg debris stored in ethanol for DNA extraction were unsuccessful given the small size (<1 mm) and static charges. Therefore, for this proof-of-concept all samples were stored at 4 °C and directly in 180 µL of ATL lysis buffer (Cat. #1014758, QIAGEN, Hilden, Germany) used for DNA extractions. While sample storage in lysis buffer may not be ideal, its use represents a compromise between the challenges of field collection and working with the material. To ensure that storage in lysis buffer did not impact the sample and downstream genetic analyses, additional controls were included: host plant tissue only (n = 3), adult tissue (n = 3), and adult tissue + host plant (n = 3). All adults were freshly deceased from natural causes.

### 2.2. DNA Extraction and Amplification

DNA was extracted from all samples per manufacturer’s instructions for animal tissue using the Qiagen DNEasy Blood & Tissue kit with some modifications. Tissue in lysis buffer was not mechanically disrupted prior to the initial incubation. Incubation in lysis buffer and proteinase K was overnight, ~12 h. To avoid clogging the silica membrane only lysate was applied to spin columns avoiding large tissue debris like that from host plant material. A single final elution 50 µL was performed. Total DNA from the resulting extracts was quantified using the high sensitivity Qubit dsDNA assay (Invitrogen, Carlsbad, CA, USA) per manufacture’s instructions with 2 µL of extract. Aliquots of DNA were normalized to the lowest total DNA concentration with molecular grade water prior to PCR. This was to ensure as much similar starting material as possible for amplification.

A 640 bp fragment of the barcoding gene COI was amplified in PCR using Lepidoptera specific primers from Hebert et al. [17]: LEP-F1, 5′-ATTCAACCAATCATAAAGATAT-3′; and LEP-R1, 5′-TAAACTTCTGGATGTCCAAAAA-3′. The COI barcoding gene was chosen because reference sequences were available for vouchered specimen of *C. thomasi bethunebakeri* enabling us to confidently confirm species identity via sequencing. Additionally, COI is one of the most commonly sequenced barcoding genes in animals so it will therefore be a versatile marker for similar research using tissue of possibly unknown origin where species identity needs to be confirmed. A master mix PCR cocktail was prepared for carrying out 20 µL reactions, each containing 1 unit of Platinum Taq DNA Polymerase (Invitrogen), 1× PCR Buffer, 2.0 mM of MgCl_2_, 0.4 mM dNTPs, 0.2 µM of each primer, 13.4 µL of PCR-grade H_2_O, and 2 µL of DNA template normalized to 1.1 ng/µL. Two reactions contained PCR-grade H_2_O in lieu of template to serve as negative controls. Thermocycling conditions were modified from Hebert et al. [17] and consisted of one cycle of 1 min at 94 °C, six cycles of 30 s at 94 °C, 40 s at 48 °C, and 1 min at 72 °C, followed by 42 cycles of 30 s at 94 °C, 40 s at 51 °C, and 1 min at 72 °C, with a final extension step of 1 min at 72 °C.

A 5 µL aliquot of PCR products was combined with 1 µL of 5× loading dye (Bioline) for Agarose gel electrophoresis. Product–dye mix was run for 120 min at 100 V alongside 5 µL HyperLadder™ 50 bp (Bioline) on a 2% TAE Agarose gel stained with Ethidium Bromide. Additionally, PCR products were quantified using the broad range Qubit dsDNA assay (Invitrogen) per manufacture’s instructions and 2 µL of product.

### 2.3. Validation

To quantitatively assess DNA extraction and gene amplification yields and capture methodological variation, mean differences in dsDNA recovered and amplicon abundance were tested for using an analysis of variance (*aov* {stats}) with Tukey’s Honest Significant Difference (*TukeyHSD* {stats}) post hoc test in R version 3.4.1 [23].

Identity of the resulting amplicons was validated via Sanger sequencing. A single exemplar PCR product from each treatment combination (tissue and storage time; n = 9; Table S1) was sent to Genewiz (Plainfield, NJ, USA) for purification and sequencing in the forward and reverse directions. Consensus sequences were generated from successfully sequenced and quality trimmed amplicons using Geneious v. 11.1.5 [24]. Gene and species identity were confirmed via NCBI’s BLASTn megablast [25] and Genbank [26], which contains COI sequences from vouchered specimens of *C. thomasi* (KY412475.1).

### 2.4. Field Testing

The efficacy of these methods with natural samples was tested by sampling egg cases of frosted elfin butterfly (*Callophrys irus irus*) populations. Citizen scientists were recruited, trained, and subsequently conducted the collection of all field samples. Participating individuals were experienced and knowledgeable butterfly watchers. Field and classroom training was provided for egg detection, collection, and storage along with a simple written protocol. A total of 84 egg case samples were collected from 23 locations in Apalachicola National Forest and Blackwater River State Forest in Florida, USA during March 2018. Collection sites were distributed across six burn units and, if possible, egg case collections were done in multiple sites within a burn unit. Sampling across and within burn units was conducted because each unit has a different management history, potentially impacting organism abundance and gene flow. In most cases, egg cases were collected from multiple plants found in the same patch of host and combined into a single sample. Pooling of egg cases within a host plant patch was performed to increase target tissue abundance and the chances of recovering target DNA. The number of eggs in each sample ranged from 2 to 20. Upon collection, the eggs were immediately preserved in ATL lysis buffer (QIAGEN, Cat. #1014758) and stored at −20 °C until DNA extraction.

Extraction and COI gene amplification were carried out as described for the proof-of-concept with *C. thomasi bethunebakeri*. Unlike for the proof-of-concept, no treatments for storage time or non-target tissue were carried out. Correlation between quantity of egg case starting material and gDNA yield was tested for using Pearson’s product-moment correlation as computed in R using *cor* {stats} and *cor.test* {stats}. For samples that failed to amplify following this methodology, additional amplification reactions were preformed using the universal COI primers LCO1490: 5′-GGTCAACAAATCATAAAGATATTGG-3′ and HC02198 5′-TAAACTTCAGGGTGACCAAAAA ATCA-3′ designed by Former et al. [27] and the same cycling conditions described for the Lepidoptera specific primers used for the proof-of-concept above. These primers were designed to amplify a 710-bp region of COI across invertebrates. All PCRs contained 2.l µL template DNA, 0.2 µM of each primer, 0.5 µg/µL of bovine serum albumin (BSA), 10 μL of 2× OneTaq Hot Start Quick-Load Master Mix (New England BioLabs, Ipswich, MA, USA) in a total volume of 20 μL. PCR products were analyzed by gel electrophoresis, purified, and sequenced via Sanger sequencing at Eurofins Genomics (Louisville, KY, USA).

Alignment and editing of resulting sequence data were performed in Geneious v. 11.1.5. To confirm species identity and eliminate non-target sequences, sequences were queried against the GenBank nucleotide database. These validated sequences were retained for downstream analyses if there was a minimum of 99% similarity over at least 96% of the query sequence to the *C. irus* reference sequence.

In order to assess population structure and connectivity additional loci with polymorphic sites were needed. Therefore, the nuclear gene elongation factor 1 alpha (EF1) was also amplified for all samples with validated COI sequences. Using primers ef44 5′-GCYGARCGYGARCGTGGTATYAC-3′ and efrcM4 5′-ACAGCVACKGTYTGYCTCATRTC-3′ [28] a 1064-bp fragment of EF1 was targeted. PCR for EF1 was carried out under the following conditions: 94 °C for 2 min; 40 cycles of 94 °C for 30 s, 58 °C for 40 s, and 68 °C for 90 s; and 68 °C for 5 min. Reactions were the same as described for COI PCR above. Because there was no reference sequence for *C. irus* EFI, only sequences matching the closely related *Ahbergia korea* with of 98% similarity over at least 96% query were retained for downstream analyses.

Sequences passing our sequence similarity quality filters for both COI and EF1 were used to obtain a coarse overview of genetic variation and population structure by conducting an analysis of molecular variance (AMOVA) in R (*poppr.amova* {poppr}). Burn unit and then locality were defined as the nested hierarchy for the AMOVA. Statistical significance of variance components was computed with a permutation test using *randtest* {ade4} and 999 permutations.

## 3. Results

### 3.1. DNA Extraction and Amplification

**Figure 2:**
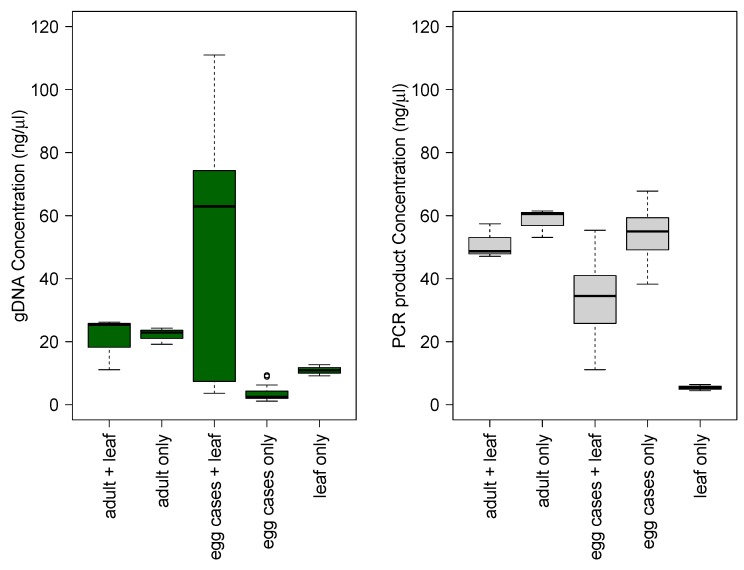
Boxplots of median, lower, and upper quartiles of genomic DNA concentration and PCR product concentration for proof-of-concept tissue treatments: adult + leaf (n = 3; positive control), adult only (n = 3; positive control), egg cases + leaf (n = 15), egg cases only (n = 15), and leaf only (n = 3, negative control). Because there was no significant difference in yield between number of storage days for egg cases + leaf and egg cases only (F = 3.864, *p* = 0.0569) all storage day treatments were pooled within tissue type treatment.

DNA was successfully extracted from all samples and all treatment types (Figure 2). Across all treatments, DNA extract concentration ranged from 1.1 to 111.0 ng/µL with an average concentration of 25.5 ng/µL (± 31.7 SD). For treatments containing only chorion from hatched ovae, gDNA yield was considerably lower ranging from 1.1 to 9.4 ng/µL with an average concentration of 3.7 ng/µL (± 2.6 SD). Even with low DNA yields, amplification using the Lepidoptera specific COI primers was successful for all samples except for reactions containing only leaf tissue (Figure 2).

### 3.2. Validation

There was no significant difference in total DNA (F = 3.864, *p* > 0.05) and target amplicon concentration (F = 0.016, *p* > 0.9) based on number of storage days therefore storage day treatments were pooled for comparisons of yields from different tissue types for subsequent analyses of variance and post-hoc tests. Genomic DNA concentration varied among tissue treatments (F = 7.28, *p* < 0.001), but only significantly so between samples containing hatched ovae only (egg cases) and hatched ovae with leaf tissue (*p* < 0.0001). Despite having the same concentration of initial template material (1.1 ng/µL) in each reaction, amplicon yields were more variable for samples containing chorion ($\bar{x}$ = 27.8 ± 35.7 SD) than those containing adult tissues ($\bar{x}$ = 21.5 ± 5.7 SD; Figure 2). There were significant differences in amplicon concentration (F = 22.15, *p* < 0.0001), primarily between the leaf tissue only product, which served as a negative control and did not successfully amplify or sequence, and all other treatments (*p* < 0.001). The egg case + leaf amplicon concentration was also significantly less than either the adult only (*p* < 0.001) and egg case only (*p* < 0.0001) amplicon concentration.

After trimming poor quality sites at amplicon termini, sequence length ranged from 537 to 600 bases for all sequencing products containing Lepidoptera tissue. Average percentage of sites with a Phred score greater than 40 (base call accuracy of 99.99%) was high, ranging from 85–100% for consensus COI sequences. Nucleotide base composition of the forward and reverse consensus sequences was identical for the eight exemplar amplicons sequenced, excluding the unsequenceable negative control containing leaf tissue only (Table S1). All sequences were a 99% match to the *C. thomasi* voucher sequence on NCBI Genbank across 100% of the query sequence. Three sites differed from the *C. thomasi* voucher sequence on NCBI Genbank.

### 3.3. Field Testing

**Figure 3:**
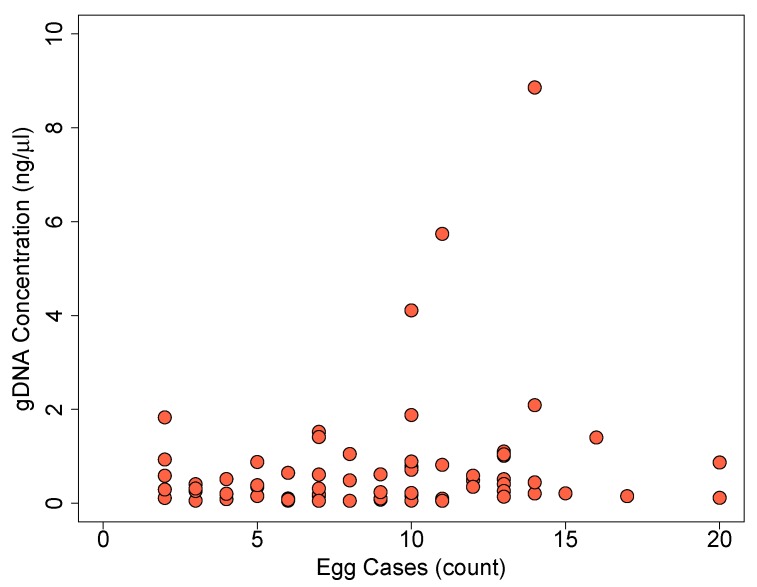
Relationship of genomic DNA yield and the number of egg cases per field collected samples (n = 79) of *C. thomasi bethunebakeri*. There was no statistical correlation between DNA concentration and the number of egg cases (r = 0.180, *p* = 0.138).

DNA was extracted from 84 field samples. Of these extracts, DNA was successfully recovered and quantified from samples containing as few as two egg cases. Genomic DNA concentration of 69 DNA extracts ranged from 0.051 ng/μL to 8.86 ng/μL and 15 extracts were below the quantification assay’s detection limits. There was no statistically significant correlation between genomic DNA concentration and the number of egg cases per sample (Figure 3, r = 0.180, *p* > 0.1).

Amplification of template DNA using COI primers and subsequent sequencing was successful for 71 extracts (84.5%), including 13 extracts that contained undetectable quantities of DNA. After quality trimming, the species identity of 58 consensus sequences was confirmed via BLAST. This final set of cleaned and validated COI sequences included eight samples that had DNA concentration below detection. The nuclear gene EF1 was successfully amplified for 31 of the 58 samples with species validated COI sequences. Amplicons from 27 EF1 PCR products were successfully sequenced and had quality trimmed consensus sequences that passed our quality filter of a 98% shared sequence similarity via BLAST with the EF1 gene sequence from *A. korea*.

The region of COI sequenced contained very little genetic variation with only three sites in 660 that varied among the 71 samples. The variant site at the third base position was only sequenced in five samples and had a minor allele frequency of 40%. The variant sites at base 68 and 623 were sequenced in all individuals and had minor allele frequencies of 12.6% and 1.4% respectively. What little genetic variation was observed in COI was not significantly contributing to population within or among burn units or locations (Table 1) as determined by an AMOVA. The nuclear gene EF1 was more variable with 11 variant sites across the 660 bases successfully sequenced in 27 samples. At only four variant sites were all 27 samples successfully sequenced. At two sites near the end of the read, base quality was not high enough for calling. However, at the remaining five sites chromatogram peak quality was good, but base identity was ambiguous due the presence of double peaks, possibly indicating heterozygosity or amplicons from different individuals. Excluding samples for which the base call was ambiguous or were not sequenced at a given site, average minor allele frequency was 12.5% (± 9.4 SD). Similarly to COI, there was no statisticallysignificant population structure associated with burn unit or locality at the EF1 locus (Table 1).

## 4. Discussion

To our knowledge, this study is the first to demonstrate that it is possible to extract DNA of sufficiently high quantity and quality from residual butterfly egg debris for successful gene sequencing. It additionally describes a simple, low-cost, and reliable method of collecting and storing egg samples that can be adopted for field or laboratory work as well as deployed with projects that have a larger geographic scope and/or involve citizen scientists.

While the methods used for field sample testing did not reveal statistically significant population structure, if it even exists, additional sampling, additional loci, and genotyping technologies that can better identify heterozygosity will improve the methods utility. Even though resolving population structure would require an expanded study design as suggested, at a minimum, the presence of a rare species can be confirmed using our sampling methods and COI DNA barcoding. Likely, much of the variation observed in sequencing success rates and DNA extraction yields has to do with how degraded the sample had become prior to being collected and stored in lysis buffer. Sample DNA degradation could be evaluated in advance of PCR using agarose gel electrophoresis or spectrophotometry. However, often DNA yields are so low that gDNA gels would exhaust the available extract and specialized equipment such as the NanoDrop spectrophotometer for evaluating degradation with less material might not be available. Proceeding directly to PCR and sequencing is likely the most effective and accessible means for testing material quality. For example, EF1 sequencing of field collected samples had poor success, likely due to degraded sample DNA. Both the large gene fragment size and the pooled nature of samples may have also contributed to poor sequencing success. However, for both genes, PCR optimization and collecting fewer egg cases per sample can likely improve sequencing success rates. When age and level of sample degradation is unknown, our field testing proves that collecting useable genetic data is possible with as few as two egg cases. From the proof-concept we show that sequencing can be successful from samples with gDNA concentrations as low as 1.1 ng/µL even when carry-over non-target host plant tissue is present. Additionally, both the proof-of-concept and field testing demonstrate the usefulness and success of sample storage directly in lysis buffer.

Our results validate that this procedure is effective and has potential broad applicability for insect population and conservation research, not only for rare or at-risk (e.g., imperiled, threatened or endangered) insect taxa, but also common unthreatened species too. Similarly, the use of residual egg debris has advantages over other documented noninvasive genetic sampling methods involving larval exuviae, frass, or the more traditional removal of tissue samples (e.g., palps, wing clippings, legs) from adult organisms. These can be problematic logistically as they often require longer organism holding times, necessitate organism removal from the environment, or may result in a limited number of viable available samples [18,19,20,21,22]. For example, Saarinen et al. [13] demonstrated that wing clips represent a viable, non-lethal method of obtaining DNA from a federally endangered butterfly. This technique however required temporary organism capture and handling as well as a sufficient number of adult organisms readily available for sampling. Similarly, Monroe et al. [11] and Scriven et al. [22] evaluated the effectiveness of using insect feces with somewhat mixed results. In both cases, the target organisms additionally needed to be captured and temporarily maintained in captivity to successfully obtain samples. While Hamm et al. [14] demonstrated that the removal of small amounts of hind wing material had no significant impact on butterfly behavior or survival, the authors do suggest that wing clipping may not be appropriate for taxa with higher wing loading. Such methodologies may additionally be unavailable due to factors including permit restrictions that might be in place to limit organism handling or mitigate potential injury (i.e., mortality, damage, or stress). The collection of residual egg debris by contrast requires no living organism contact, and thereby would not potentially compromise small populations of rare or endangered species while at the same time allowing adequate sample sizes for population-level genetic analysis. It furthermore offers a potentially large pool of available samples that can be obtained over a somewhat longer time period than simply the phenology of a particular developmental stage or under weather conditions such as cloudy skies or cool temperatures that would typically not be optimal for adult activity and collection. This in turn would help provide added sample collection flexibility and/or opportunity.

The simplicity of the sample collection and storage protocol facilitates the use of trained citizen scientists to conduct field sampling as was done for this study, thereby significantly increasing both the project scope and the potential total number of samples collected. While volunteer training for field survey and collection protocols was necessary, the time commitment was minimal requiring just 1–1.5 days. This is substantially less in our opinion than what would be necessary to reach the appropriate skill level for adult collection, handling, and sampling while ensuring appropriate safeguards.

Residual egg debris sample collection is not without potential limitations. It is inherently labor-intensive owing to the small size of egg cases and their overall limited detectability in the larger landscape. Detailed ecological, life history, and behavioral knowledge of the target organism such as oviposition behavior and preference can help identify priority search areas and enhance detection probability in the field. In the case of *Callophrys irus irus*, the encounter rate of adult butterflies in Florida populations is generally low and quite limiting for nondestructive tissue sample collection. Egg and larval abundance by contrast tends to be locally high. Habitat access or impact may also be a factor. For egg debris collection to be effective, extensive larval host plant examination is required. This could potentially result in negative impacts to sensitive habitat areas such as trampling or disturbance to other rare taxa. All of these factors of course vary tremendously from one taxon to another, necessitating a highly targeted approach tailored to the specific organism and circumstance.

Sample degradation is also a potential concern. As the proof-of-concept indicates, samples known to be fresh when collected guarantee the best success for generating genetic data. However, even when there is uncertainty surrounding tissue and collection conditions, this study demonstrates that generating useable genetic data is possible even from samples with DNA yields below detection limits. Likely, much of the variation observed in the DNA extraction and sequencing success of field samples has to do with how the degraded the sample had become prior to being collected and stored in lysis buffer. It is unknown how long residual egg debris can reside in the environment and remain viable for DNA extraction and analysis. This undoubtedly depends on many factors, including total duration time, time of year, geographic location, sample location, ambient temperature, light intensity, etc. Additional trails are needed to better understand the potential temporal limitations of this nondestructive genetic sampling technique and sample source.

## 5. Conclusions

Our study demonstrated that it is possible to extract DNA of sufficiently high quantity and quality for successful single gene sequencing from the chorion of residual butterfly egg debris left behind from recently hatched larvae. We tested the potential impact of non-target tissue carryover by comparing samples with egg cases only versus those with egg cases plus leaf tissue and found that even with low DNA yields, amplification using the Lepidoptera specific COI primers was successful for all samples. Additionally, we describe a simple, low-cost, and reliable method of collecting and storing egg debris samples. This effective, nondestructive technique has potential broad applicability for insect population and conservation research, including for projects that have a larger geographic scope and/or involve trained citizen scientists. Potential limitations include a lack of egg case detectability in the field, increased labor to secure an adequate number of samples, and sample degradation. Additional rigorous evaluation is needed to fully determine the strengths and weaknesses of this and other noninvasive genetic sampling techniques for research.

## Figures and Tables

**Table 1:** Hierarchical test of molecular variance to test for genetic variation in COI (n = 58) and EF1 (n = 27) genes associated with burn unit and locality for field collected egg case samples of *C. thomasi bethunebakeri.*

Gene	Source of Variation	DF	Variance	Variance (%)	*p*-Value
COI	Among burn units	6	0.0189	6.2	0.336
	Among locations within burn units	11	0.0012	0.4	0.760
	Within locations	40	0.2842	93.4	0.285
	Total	57	0.3043		
EF1	Among burn units	6	0.0385	4.0	0.436
	Among locations within burn units	10	0.0898	9.4	0.403
	Within locations	10	0.8321	86.6	0.279
	Total	26	0.9603		

## Supplementary Materials

The following are available online at https://www.mdpi.com/2075-4450/10/10/311/s1, Table S1: Proof-of-concept sequencing and blast results.
