# Supplementary material for: Using Noninvasive Genetic Sampling to Survey Rare Butterfly Populations
Source: Insects. 2019 Sep 23;10(10):311. doi: 10.3390/insects10100311 (PMC6835262; doi:10.3390/insects10100311)
Supplement: Supplementary file 1 [file insects-10-00311-s001.pdf]

Supplementary Table 1. Sanger sequencing and NCBI BLAST results for exemplar amplicons from each treatment of the proof-of concept study

Sample Code	Tissue	Storage Treatment	Sequence Quality[1]	Sequence Length[2]	Top Hit	Accession	e-value	Query Coverage	Identity
Ct003	egg cases only	1 day	100.0	600	*Cyclargus thomasi*	KY412475.1	0	100%	99%
Ct008	egg cases only	5 days	100.0	600	*Cyclargus thomasi*	KY412475.1	0	100%	99%
Ct013	egg cases only	14 days	100.0	600	*Cyclargus thomasi*	KY412475.1	0	100%	99%
Ct018	egg cases + leaf	1 day	100.0	600	*Cyclargus thomasi*	KY412475.1	0	100%	99%
Ct022	egg cases + leaf	5 days	92.5	545	*Cyclargus thomasi*	KY412475.1	0	100%	99%
Ct026	egg cases + leaf	14 days	89.9	537	*Cyclargus thomasi*	KY412475.1	0	100%	99%
Ct031	adult	NA	100.0	598	*Cyclargus thomasi*	KY412475.1	0	100%	99%
Ct034	adult + leaf	NA	100.0	600	*Cyclargus thomasi*	KY412475.1	0	100%	99%
Ct038	leaf only	NA	26.9	367	*NA*	NA	NA	NA	NA

[1] Sequence quality is the average percentage of sites with a Phred score greater > 40 (base call accuracy of 99.99%).[2] Sequence length is the quality trimmed consensus sequence length.
